# Semaglutide Protects against 6-OHDA Toxicity by Enhancing Autophagy and Inhibiting Oxidative Stress

**DOI:** 10.1155/2022/6813017

**Published:** 2022-07-13

**Authors:** Dong-xing Liu, Chen-sheng Zhao, Xiao-na Wei, Yi-peng Ma, Jian-kun Wu

**Affiliations:** Department of Neurology, Shanxi Cardiovascular Hospital, Taiyuan, Shanxi, China

## Abstract

Parkinson's disease (PD) is the second most prevalent neurodegenerative disorder for which no effective treatment is available. Studies have demonstrated that improving insulin resistance in type 2 diabetes mellitus (T2DM) can benefit patients with PD. In addition, a neuroprotective effect of glucagon-like peptide-1 (GLP-1) receptor agonists was demonstrated in experimental models of PD. In addition, there are some clinical trials to study the neuroprotective effect of GLP-1 analog on PD patients. Semaglutide is a long-acting, once-a-week injection treatment and the only available oral form of GLP-1 analog. In the present study, we treated the human neuroblastoma SH-SY5Y cell line with 6-hydroxydopamine (6-OHDA) as a PD in vitro model to explore the neuroprotective effects and potential mechanisms of semaglutide to protect against PD. Moreover, we compared the effect of semaglutide with liraglutide given at the same dose. We demonstrated that both semaglutide and liraglutide protect against 6-OHDA cytotoxicity by increasing autophagy flux and decreasing oxidative stress as well as mitochondrial dysfunction in SH-SY5Y cells. Moreover, by comparing the neuroprotective effects of semaglutide and liraglutide on PD cell models at the same dose, we found that semaglutide was superior to liraglutide for most parameters measured. Our results indicate that semaglutide, the new long-acting and only oral GLP-1 analog, may be represent a promising treatment for PD.

## 1. Introduction

Parkinson's disease (PD) is a neurodegenerative pathology that leads to delayed movement, quiescent tremor, myotonia, slow faltering gait, and imbalance [1]. The death of millions of people worldwide results from PD [2]. However, the current drugs for treating PD can only relieve symptoms without affecting disease progression. Furthermore, long-term complications are associated with obvious adverse reactions. Thus, more efficacious and safer drugs to treat PD are required. Recently, epidemiological studies demonstrated an increased risk of PD in patients with type 2 diabetes mellitus (T2DM) [3]. It was subsequently found that PD and T2DM share several pathological features, including insulin signaling impairment, oxidative stress, and neuroinflammation [4], suggesting that drugs used for diabetes could be used to treat PD.

The effects of glucagon-like peptide-1 (GLP-1) analogs, mediated by GLP-1R, facilitate insulin signaling and glucose homeostasis in T2DM. GLP-1R is not only expressed by pancreatic islets but also found to be localized in the brain, especially among the frontal cortex, hypothalamus, thalamus, hippocampus, cerebellum, and substantia nigra [5]. Increasing evidence show that GLP-1 analogs can cross the blood–brain barrier (BBB) and affect various cellular pathways in the central nervous system (CNS), such as neuroinflammation, mitochondrial function, and cell proliferation [6–8]. Therefore, researchers are actively investigating the effect of GLP-1 analogs on PD. Exendin-4 (exenatide, bydureon), which is clinically used for treating diabetes, has a protective effect in different animal models of PD and patients with PD, as demonstrated in a pilot clinical trial (NCT01174810) [9–11]. Importantly, a phase II clinical trial showed a protective effect on patients with PD, in which Exendin-4 arrested disease progression, even when treatment was interrupted for 3 months [12]. Liraglutide, another GLP-1 analog, was also found to have neuroprotective effects in animal models of PD [13]. A phase II clinical trial for its protective effects in patients with PD are currently underway (clinical trial identifier NCT02953665).

Semaglutide is another GLP-1 analog that resists protease degradation and extends its half-life time to allow for weekly administration [14]. Recently, the US Food and Drug Administration (FDA) approved the oral form of semaglutide [15], resulting in simpler and more convenient therapies for patients. In 2018, it was approved for the treatment of diabetes in the United States, Europe, and Canada [16]. Semaglutide has similar safety to the previous GLP-1 analogs for the treatment of diabetes, without new adverse reactions [12]. In 2019, phase II clinical trials testing the protective effect of semaglutide in patients with PD were initiated (NCT03659682). Recently, the neuroprotective outcome of semaglutide in PD mouse models was also demonstrated [17]. Therefore, we aimed to provide further insights into investigating the neuroprotective effect of semaglutide, compared with equivalent doses of liraglutide in the SH-SY5Y cell line treated with 6-OHDA.

Autophagy is a dynamic recovery process of substance self-clearing in cells. Pathological studies have found compression of proteins related to the autophagy–lysosomal pathway and reduction of lysosomal enzyme activity in patients with PD [18]. Given the onset of PD related to autophagy disorders, enhancing autophagy has great potential as a therapy for PD [19]. Studies have demonstrated that raising autophagy critically allows the GLP-1 analog to exert a wide range of effects, including neuroprotection [20, 21]. In addition, through continuous research, oxidative stress and mitochondrial dysfunction were found to be involved in the pathogenesis of PD by mediating apoptosis [22, 23]. Therefore, we suggest that the enhancement of autophagy and antioxidative stress, together with a reduction in mitochondrial dysfunction, are pivotal molecular mechanisms underlying the beneficial outcomes of semaglutide against 6-OHDA-mediated toxicity. Dopaminergic neurons, which are mainly affected in PD, are difficult to gain and maintain. Therefore, we chose the SH-SY5Y cell line for our research, which is related to its human origin and dopaminergic-like neuronal properties [24]. In our work, we have examined the molecular mechanisms behind the protective effect of semaglutide on 6-OHDA-treated SH-SY5Y cells in vitro, focusing on autophagy and oxidative stress as well as mitochondrial dysfunction.

## 2. Materials and Methods

### 2.1. Chemicals and Reagents

The SH-SY5Y cell line of human neuroblastoma was collected from the Stem Cell Bank of the Chinese Academy of Sciences. Semaglutide (peptide purity: 95.77%) and liraglutide (peptide purity: 95.77%) were obtained from Synpeptide Co. (Shanghai, China); 6-OHDA and the anti-LC3-II antibody were obtained from Sigma-Aldrich (St. Louis, MO, USA); the anti-P62 antibody, anti- Atg7 antibody, and anti-beclin1 antibody were obtained from Abcam (Cambridge, UK); and the *β*-actin antibody was obtained from Bioworld Technology Co. (Shanghai, China). The reactive oxygen species (ROS) assay kit and mitochondrial membrane potential assay kit with JC-1 were purchased from Nanjing KeyGen Biotechnology Company (Nanjing, China).

### 2.2. Cell Culture

The SH-SY5Y cells were grown in DMEM/F-12 medium containing 10% heat-inactivated FSB and penicillin–streptomycin (100 U/mL) and then placed in a humidified incubator with 5% CO_2_ at 37°C. The cells were subsequently cultured in 96-well plates or 6-well plates at a density of 5 × 10^4^ cells/well for the future experiment.

### 2.3. Cell Viability Assay

The cell viability was measured using MTT assay. To determine the appropriate concentration of 6-OHDA, the SH-SY5Y cells were treated for 24 h with 0, 25, 50, 75, 100, or 125 *μ*M 6-OHDA, and their viability was measured using the MTT assay. Considering that the survival rate of cells significantly decreased with 75, 100, or 125 *μ*M/L 6-OHDA, 75 *μ*M 6-OHDA was selected as the optimal concentration for the experiments. Similarly, MTT was used to determine the viability of SH-SY5Y cells treated with 0, 1, 10, and 100 nM semaglutide or liraglutide combined with or without 75 *μ*M 6-OHDA for 24 h. The concentration of semaglutide and liraglutide was selected on the basis of previous studies [25–27]. The survival rate of SH-SY5Y was calculated using the common formula (an experimental group/a control group × 100%).

### 2.4. Western Blot

SH-SY5Y cells were lysed with RIPA protein lysis buffer (Beyotime Biotechnology, Shanghai, China) for 30 min. After centrifugation, proteins in the supernatant were extracted and measured using the BCA protein proof (Beyotime Biotechnology, Shanghai, China). Equal amounts of protein (50 *µ*g) per lane were loaded onto a 10% sodium dodecyl sulfate polyacrylamide gel electrophoresis (SDS-PAGE) (Beyotime Biotechnology, Shanghai, China) and transferred to a polyvinylidene fluoride (PVDF) membrane (Beyotime Biotechnology, Shanghai, China). The PVDF membranes were blocked for 2 h with 5% skimmed milk in Tris-buffered saline tween (TBST) at room temperature and then incubated overnight with anti-LC3-II, anti- P62, anti-beclin1, and anti-Atg7 antibodies at 4°C. The membrane was incubated with horseradish peroxidase-conjugated secondary antibody at room temperature for 2 h. The protein bands were visualized using enhanced chemiluminescence with *β*-actin as an internal control. Quantity One (Bio-Rad) and Image-Pro were used for analysis.

### 2.5. Measurement of Reactive Oxygen Species Levels

Intracellular ROS production was measured by flow cytometry using dichlorodihydrofluorescein diacetate (DCFH-DA) as previously described, with slight modification [28]. Briefly, after cells were harvested, the cells were stained with 10 *μ*M DCFH-DA for 30 min at 37°C in the incubator. Cells were collected, and dihydrodichlorofluorescein (DCF) fluorescence was analyzed by a BD FACSCalibur flow cytometer (BD Biosciences, Franklin Lakes, NJ, USA). The percent fluorescence intensity (%) = the mean fluorescence of each group/the mean fluorescence of negative control group × 100.

### 2.6. Measurement of Mitochondrial Membrane Potential

Mitochondrial membrane potential was analyzed via flow cytometry using JC-1 according to the instructions of the assay kit. Briefly, after treatment, the cells were stained with a JC-1 working buffer for 20 min at 37°C in the incubator. Then, they were harvested, centrifuged, and resuspended in the incubation medium. Fluorescence intensity was determined via fluorescence spectroscopy (F-2700 Techcomp (China) Ltd.). The relative mitochondrial membrane potential was evaluated by calculating the ratio of red/green fluorescence intensity.

### 2.7. Statistical Analyses

Experimental data were analyzed using SPSS 19.0 and GraphPad Prism 6.0. All data are shown as the mean ± standard deviation (SD). One-way ANOVA followed by post hoc Tukey's test was used to compare the differences among multiple groups, and LSD *t*-test was used to compare the differences between two groups. *P*-values less than 0.05 were considered statistically significant.

## 3. Results

### 3.1. Semaglutide and Liraglutide Reverse Loss of Cell Viability Induced by 6-OHDA in SH-SY5Y Cells

**Figure 1:**
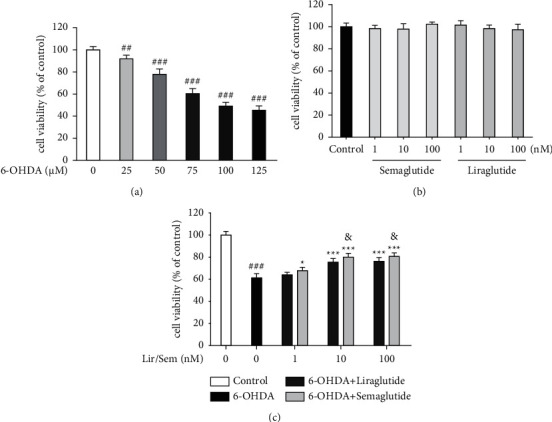
(a) With the increase of 6-OHDA concentration, the survival rate of SH-SY5Y cells decreased gradually. And the survival rate of SH-SY5Y cells was 50% of the control group when the 6-OHDA concentration was 75 *μ*mol/L. ^###^*P* < 0.001, ^##^*P* < 0.01 vs. control. (b) Different concentrations of semaglutide and liraglutide had no significant effect on the survival rate of SH-SY5Y cells compared with the control group. (c) Semaglutide and liraglutide reverse cytotoxicity in 6-OHDA-treated SH-SY5Y cells, whereas semaglutide is more effective. Cells were treated with 6-OHDA (75 *μ*M) for 24 (h). ^###^*P* < 0.001 vs. control. ^*∗*^*P* < 0.05, ^*∗∗*^*P* < 0.01, ^*∗∗∗*^*P* < 0.001 vs. 6-OHDA treatment. ^&^*P* < 0.05 compared with the 6-OHDA + liraglutide group. An MTT assay was employed to measure cell viability. Data was expressed as mean ± standard deviation.

The results indicated that 6-OHDA exerts a concentration-dependent effect on cell viability (Figure 1(a)). The 6-OHDA (75 *µ*M) treatment significantly decreased cell viability to 60.5% ± 4.5% compared with the control (100% ± 3.1%). On this basis, 6-OHDA at a concentration of 75 *μ*M was selected for further studies.

Compared with the untreated control, no significant differences were observed in the viability of SH-SY5Y cells receiving semaglutide or liraglutide (Figure 1(b)). As presented in Figure 1(c), semaglutide and liraglutide prevented the toxicity of 6-OHDA. The survival rates of SH-SY5Y cells increased from 60.5% ± 4.5% upon treatment with 75 *µ*M 6-OHDA to 64.1% ± 2.3%, 75.4% ± 3.3%, and 76.2% ± 3.5% after treatment at different concentrations (1, 10, and 100 nM, resp.) of liraglutide. They further reached 67.7% ± 3.2%, 80.1% ± 3.4%, and 80.8% ± 3.1% after treatment at different concentrations (1, 10, and 100 nM, resp.) of semaglutide. No significant difference was observed in cell survival between the 10 and 100 nM concentrations. Consequently, the 10 nM concentration of semaglutide and liraglutide was selected for further studies. In addition, comparison between the two groups at the same concentration revealed that semaglutide was significantly more effective than liraglutide.

### 3.2. Semaglutide and Liraglutide Protect against 6-OHDA Toxicity by Enhancing Autophagy

**Figure 2:**
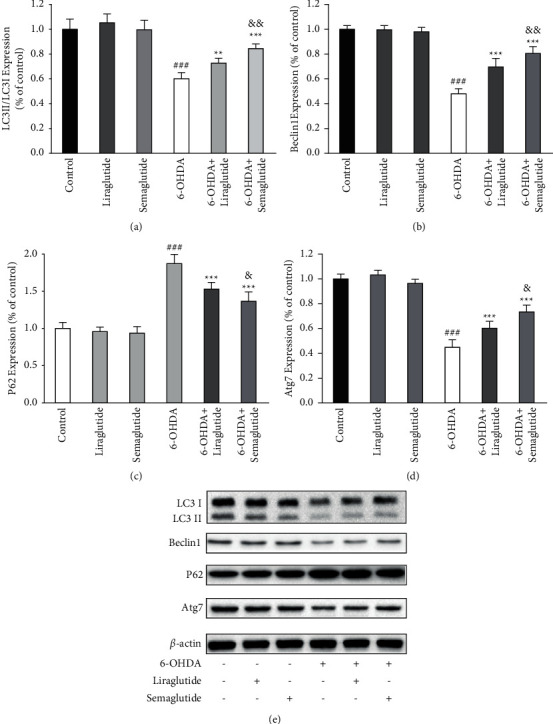
The effect of semaglutide and liraglutide on autophagy-related protein expression in SH-SY5Y cells with 6-OHDA treatment. (a) LC3-II/LC3-I ratio was downregulated in the 6-OHDA group (^###^*P* < 0.001 vs. the control group). Semaglutide and liraglutide normalized LC3-II/LC3-I ratio (^*∗∗*^*P* < 0.01, ^*∗∗∗*^*P* < 0.001 vs. 6-OHDA group) and semaglutide was significantly more effective (^&&^*P* < 0.01 vs. 6-OHDA + liraglutide group). (b) Beclin-1 expression was downregulated in the 6-OHDA group (^###^*P* < 0.001 vs. the control group). Semaglutide and liraglutide normalized beclin-1 expression (^*∗∗∗*^*P* < 0.001 vs. 6-OHDA group) and semaglutide was significantly more effective (^&&^*P* < 0.01 vs. 6-OHDA + liraglutide group). (c) P62 expression was upregulated in the 6-OHDA group (^###^*P* < 0.001 vs. the control group). Semaglutide and liraglutide normalized P62 expression (^*∗∗∗*^*P* < 0.001 vs. 6-OHDA group) and semaglutide was significantly more effective (^&^*P* < 0.05 vs. 6-OHDA + liraglutide group). (d) Atg7 expression was downregulated in the 6-OHDA group (^###^*P* < 0.001 vs. the control group). Semaglutide and liraglutide normalized Atg7 expression (^*∗∗∗*^*P* < 0.001 vs. 6-OHDA group) and semaglutide was significantly more effective (^&^*P* < 0.05 vs. 6-OHDA + liraglutide group). (e) Western blots of LC3-II, LC3-I, beclin-1, p62, and Atg7 expression. All data were expressed as mean ± standard deviation (*n* = 4).

6-OHDA treatment decreased the relative levels of LC3-II/LC3-I, beclin1, and Atg7 from 100% ± 7.2%, 100% ± 2.5%, and 100% ± 3.1% (control group) to 60.3% ± 5.0%, 48.3% ± 4.1%, and 45.1% ± 6.2% and increased the relative levels of p62 from 100% ± 8.3% (control group) to 187.2% ± 12.4% (Figures 2(a)–2(d)), indicating that 6-OHDA could inhibit autophagy of SH-SY5Y cells. However, there was no change in the levels of LC3-II/LC3-I, beclin1, p62, and Atg7 in liraglutide group (105.3% ± 7.1%, 99.7% ± 3.3%, 95.9% ± 6.0%, and 103.3% ± 3.8%) and in semaglutide group (99.7% ± 7.6%, 98.5% ± 3.4%, 93.7 ± 8.9%, and 96.4% ± 3.4%) compared with control group (Figures 2(a)–2(d)), indicating that liraglutide and semaglutide could not affect autophagy in normal SH-SY5Y cells.

In 6-OHDA + liraglutide group, the levels of LC3-II/LC3-I, beclin1, p62, and Atg7 were 72.8% ± 3.9%, 65.97% ± 6.7%, 153.1% ± 8.8%, and 60.2% ± 5.7%, respectively (Figures 2(a)–2(d)). In 6-OHDA + semaglutide group, the levels of LC3-II/LC3-I, beclin1, p62, and Atg7 were 84.5% ± 3.6%, 80.6% ± 5.5%, 136.6% ± 12.5%, and 74.3% ± 5.5%, respectively (Figures 2(a)–2(d)). Statistical analysis found that, in 6-OHDA-treated SH-SY5Y cells, both liraglutide and semaglutide could increase the levels of LC3-II/LC3-I, beclin1, and Atg7 and decrease the level of p62, indicating that semaglutide and liraglutide partially reversed the inhibitory effect of 6-OHDA on autophagy. Comparison of the 6-OHDA + liraglutide and 6-OHDA + semaglutide group also revealed that semaglutide was significantly more effective in regularizing autophagy than liraglutide in 6-OHDA-treated SH-SY5Y cells.

### 3.3. Semaglutide and Liraglutide Protect against 6-OHDA by Inhibiting ROS

**Figure 3:**
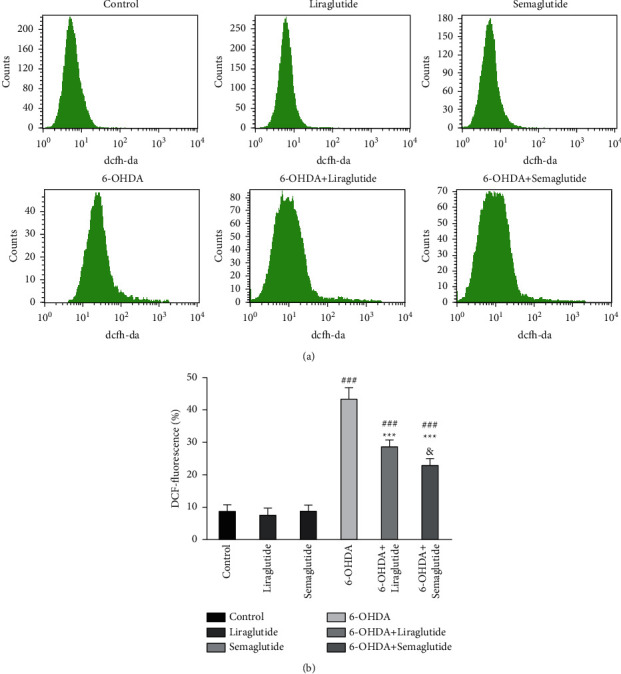
Changes in ROS production in SH-SY5Y cells. (a) Detection of ROS by flow cytometry. (b) ROS expression was upregulated in the 6-OHDA group (^###^*P* < 0.001 vs. the control group). Semaglutide and liraglutide normalized ROS expression (^*∗∗∗*^*P* < 0.001 vs. 6-OHDA group) and semaglutide was significantly more effective (^&^*P* < 0.05 vs. 6-OHDA + liraglutide group). All data were expressed as mean ± standard deviation (*n* = 4).

Flow cytometry revealed that exposing SH-SY5Y cells to 6-OHDA significantly increased their intracellular ROS levels contrary to unexposed control (43.30% ± 3.56% vs. 8.71% ± 2.04%) (Figures 3(a) and 3(b)). However, semaglutide and liraglutide alone did not influence the ROS levels in SH-SY5Y cells (8.79% ± 1.88%, 7.56% ± 2.18%) compared with the control group (Figures 3(a) and 3(b)). In contrast, semaglutide and liraglutide blunted ROS levels induced by 6-OHDA, with the ROS levels dramatically decreasing to 22.90% ± 2.12% and 28.65% ± 2.11% in SH-SY5Y cells treated with semaglutide or liraglutide and 6-OHDA (Figures 3(a) and 3(b)). Comparison of the liraglutide and semaglutide groups revealed that semaglutide was more effective in mitigating ROS levels than liraglutide.

### 3.4. Semaglutide and Liraglutide Protect against 6-OHDA by Inhibiting Mitochondrial Membrane Potential (ΔΨ*m*)

**Figure 4:**
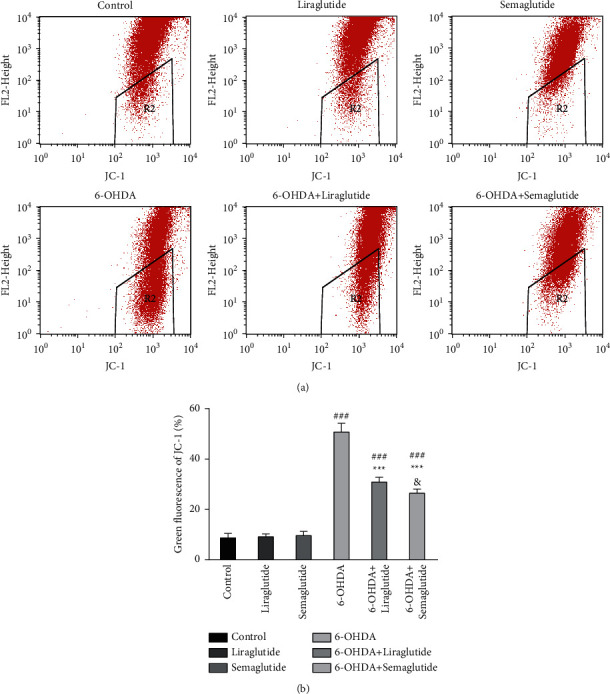
Changes in the ΔΨ*m* levels in SH-SY5Y cells. (a) Detection of the percentage of green fluorescence of JC-1 by flow cytometry. Green fluorescence indicates a low mitochondrial membrane potential (ΔΨ*m*). (b) The green fluorescence of JC-1 was upregulated in the 6-OHDA group (^###^*P* < 0.001 vs. the control group). Semaglutide and liraglutide normalized the green fluorescence of JC-1 (^*∗∗∗*^*P* < 0.001 vs. 6-OHDA group) and semaglutide was significantly more effective (^&^*P* < 0.05 vs. 6-OHDA + liraglutide group). All data were expressed as mean ± standard deviation (*n* = 4).

Red fluorescence indicates a high mitochondrial membrane potential, whereas green fluorescence indicates a low mitochondrial membrane potential. Therefore, the change in the ΔΨ*m* levels can be determined by the change of fluorescence color. Cells exposed to 75 *μ*M 6-OHDA for 24 h had a notable increase in green fluorescence (50.71% ± 3.51%) compared with control (8.72% ± 1.82%), indicating a depletion of ΔΨ*m* (Figures 4(a) and 4(b)), which is concomitant with mitochondrial dysfunction. However, semaglutide and liraglutide alone did not influence the ΔΨ*m* levels in SH-SY5Ycells (9.60% ± 1.72%, 9.19% ± 1.06%) compared with the control group (Figures 4(a) and 4(b)). Semaglutide or liraglutide treatment with 6-OHDA in SH-SY5Y cells significantly improved the ΔΨ*m* levels (26.46% ± 1.64%, 30.86% ± 1.90%), supporting the role of semaglutide and liraglutide in preventing 6-OHDA-induced mitochondrial toxicity (Figures 4(a) and 4(b)). Comparison of the liraglutide and semaglutide groups also revealed that semaglutide was more effective in enhancing ΔΨ*m* than liraglutide.

## 4. Discussion

Semaglutide is a modified version of liraglutide with a longer biological half-life [16]. Real-world effectiveness analysis proved that semaglutide is more effective than liraglutide in lowering HbA1c in people with diabetes [29]. Recently, semaglutide, the only oral GLP-1R (GLP-1 receptor) agonist, similar in action to injectable GLP-1R agonists, was approved for use in the United States by the FDA [15]. Semaglutide provided a more convenient treatment for patients. We thus investigated whether semaglutide is more protective than liraglutide, a common GLP-1 receptor agonist, in the treatment of PD. Previous experiment revealed that semaglutide has better neuroprotective properties than liraglutide in an animal model of PD [17]. Thus, we further explored the effects of semaglutide compared with liraglutide in a cell model of PD, together with the underlying molecular mechanisms.

Our research indicated that the GLP-1R analogs liraglutide and semaglutide exert neuroprotective effects against 6-OHDA-induced cytotoxicity in SH-SY5Y cells. First, the neuroprotective effects of the long-acting GIP-1 analog semaglutide were verified in this cell model of PD. The results indicated that treatment with liraglutide or semaglutide after 6-OHDA increased cell survival rate, enhanced autophagy, and reduced mitochondrial apoptosis compared with 6-OHDA alone. In addition, semaglutide was more protective than liraglutide in treating the cell model of PD.

Loss of dopaminergic neurons is a major pathological feature of Parkinson's disease, and therefore the experimental system that can study the characteristics of dopaminergic neurons is needed. In recent years, studies have started induced pluripotent stem cells (iPSCs) to differentiate into a dopaminergic neuron phenotype to generate human-derived dopaminergic neurons and have identified their susceptibility to toxicity [30, 31]. Meanwhile, studies confirmed the presence of increased ROS accumulation and impaired autophagy in them [32, 33]. This seems to be a perfect choice; however, the establishment and maintainability are very difficult. Therefore, the SH-SY5Y cell line, which is easily accessible, fast to propagate, simple to manipulate, and similar to human dopaminergic neurons in terms of cell morphology, physiology, and biochemical functions, became the most suitable choice for our research.

6-OHDA, an isolated hydroxylated analog of dopamine, which can freely enter dopaminergic neurons where it elevates oxidative stress levels and triggers apoptotic events, can reproduce the changes in basal ganglia circuitry and pharmacology in patients with PD [34]. Besides, recent studies have demonstrated that 6-OHDA could induce the accumulation of alpha-synuclein (*α*-Syn), another remarkable pathological characteristic of PD [35, 36]. Thus, 6-OHDA had been used to develop PD cell and animal models [37]. In our study, the observed effect of 6-OHDA on SH-SY5Y cells was consistent with that in previous studies. In addition, our work demonstrated that semaglutide prevents the decrease in cell viability induced by 6-OHDA in SH-SY5Y cells, which is in agreement with the finding in previous animal studies [17]. This finding confirms that semaglutide has a protective effect on the PD cell model that we examined.

Through our experiments, we confirmed that semaglutide activated the repression of autophagy induced by 6-OHDA in SH-SY5Y cells. Autophagy is a physiological process to recycle harmful organelles and proteins and maintain cellular energy and nutrient homeostasis under starvation. The accumulation of *α*-Syn is related to changes in the autophagy–lysosomal pathway damaging its function, thus resulting in a circle of neuronal death [38]. Ghavami et al. proposed that the enhancement of autophagy and the inhibition of apoptosis might serve as novel therapeutic targets for the treatment of PD [39]. The protective effect of GLP-1R agonists on PD has entered the stage of clinical research. Researchers are now exploring whether this protective effect is related to autophagy, as demonstrated in an MPTP-induced mouse model [6], SH-SY5Y cell line [40], and spinal cord injury rat model [41]. In addition, GLP-1R agonists have been identified as presenting multipotent effects in the CNS. Across several animal and cellular models of PD (MPTP, 6-OHDA, rotenone), it was reported that different GLP-1R agonists or GLP-1 mimic can promote neurogenesis, neuronal differentiation, and autophagy, as well as increasing nutritional factors, inhibiting neuronal apoptosis, dampening microglial reactivity, enhancing anti-inflammatory/antioxidant effects, and reducing *α*-Syn expression [6, 7, 9, 10, 42, 43]. Nevertheless, whether semaglutide, a drug that is safe enough and very effective, but also more convenient than other GLP-1 receptor agonists, can exert its neuroprotective effect related to the promotion of autophagy in PD had remained undetermined.

The cytoplasmic form of LC3-I is transformed to the phosphatidylethanolamine-bound form (LC3-II) and combines with the autophagosome membrane [44]. Therefore, the LC3-II/LC3-I ratio is a common label of autophagy. The degradation of p62 is widely used for monitoring autophagic activity as p62 can directly bind to LC3 and be selectively degraded during autophagy [45]. Atg7 also contributes to the autophagic conjugation system and autophagosome formation [46]. Similarly, the expression of the autophagy-related gene beclin1 is generally used to monitor the development of autophagosomes. Thus, we used the changes of these proteins to assess the level of autophagy in each group. Our findings indicate that a key molecular event involving the raising of autophagy flux was a protective response of semaglutide and liraglutide to 6-OHDA toxicity. Furthermore, compared with liraglutide, semaglutide was more effective in our study when both drugs were tested at the same concentration. Recently, experiments establishing a rat model of *α*-synucleinopathy suggested that an increase in autophagy may be the fundamental mechanism underlying the neuroprotective effect of GLP-1R agonists, which is supported by our study [8].

ROS are involved in the signal transduction of cell proliferation, differentiation, and apoptosis. Excessive ROS production leads to oxidative stress and mitochondrial permeability transition pore (mPTP) opening, further generating ROS. Afterwards, ΔΨ*m* collapses and cytochrome c is released, which further activates cytochrome c-mediated caspase family signaling and apoptosis [47]. This mechanism indicates that oxidative stress can induce early apoptosis by triggering mitochondrial dysfunction. In addition, oxidative stress is systemically present in PD. In this disease, antioxidants were found to decrease ROS levels and their harmful consequences [48]. Later, when investigating the neuroprotective effects of GLP-1R agonists on PD, researchers found that this is related to its function of antioxidative stress and reduction of mitochondrial dysfunction [7, 23]. Consistent with this, our work demonstrates that both semaglutide and liraglutide can reduce ROS levels and increase mitochondrial membrane potential in SH-SY5Y cells exposed to 6-OHDA. Semaglutide was more potent than liraglutide across our experiment.

## 5. Conclusions

In summary, our result validated the neuroprotective role of both semaglutide and liraglutide against 6-OHDA-induced neurotoxicity in SH-SY5Y cells. The beneficial effects of these two incretin peptides appear to be associated with the enhancement of autophagy, together with the inhibition of oxidative stress and mitochondrial dysfunction. In addition, our findings suggest that semaglutide has advantages compared with liraglutide in this regard. Semaglutide is currently in clinical trials for patients with PD. The result of these trials will allow for determining whether this outcome translates into the clinic with the hope of stopping disease progression.

## Data Availability

The data used to support the findings of this study are included within the article.
